# Pyrazinamide Susceptibility Is Driven by Activation of the SigE-Dependent Cell Envelope Stress Response in Mycobacterium tuberculosis

**DOI:** 10.1128/mbio.00439-21

**Published:** 2022-02-01

**Authors:** Joshua M. Thiede, Nicholas A. Dillon, Michael D. Howe, Ranee Aflakpui, Samuel J. Modlin, Sven E. Hoffner, Faramarz Valafar, Yusuke Minato, Anthony D. Baughn

**Affiliations:** a Department of Microbiology and Immunology, University of Minnesota Medical School, Minneapolis, Minnesota, USA; b West African Centre for Cell Biology of Infectious Pathogens, University of Ghana, Accra, Ghana; c Laboratory for Pathogenesis of Clinical Drug Resistance and Persistence, Biomedical Informatics Research Center, Division of Epidemiology, School of Public Health, San Diego State University, San Diego, California, USA; d Department of Global Public Health, Karolinska Institute, Stockholm, Sweden; e Department of Microbiology, Fujita Health University School of Medicine, Toyoake, Japan; Washington University School of Medicine in St. Louis

**Keywords:** cell envelope, drug discovery, drug resistance mechanisms, genomics, metabolism, tuberculosis

## Abstract

Pyrazinamide (PZA) plays a crucial role in first-line tuberculosis drug therapy. Unlike other antimicrobial agents, PZA is active against Mycobacterium tuberculosis only at low pH. The basis for this conditional drug susceptibility remains undefined. In this study, we utilized a genome-wide approach to interrogate potentiation of PZA action. We found that mutations in numerous genes involved in central metabolism as well as cell envelope maintenance and stress response are associated with PZA resistance. Further, we demonstrate that constitutive activation of the cell envelope stress response can drive PZA susceptibility independent of environmental pH. Consequently, exposure to peptidoglycan synthesis inhibitors, such as beta-lactams and d-cycloserine, potentiate PZA action through triggering this response. These findings illuminate a regulatory mechanism for conditional PZA susceptibility and reveal new avenues for enhancing potency of this important drug through targeting activation of the cell envelope stress response.

## INTRODUCTION

Mycobacterium tuberculosis, the etiological agent of tuberculosis (TB), is the leading cause of death by a single pathogen, killing 1.5 million people each year (1). Current TB short-course therapy consists of a four-drug regimen including isoniazid (INH), rifampicin (RIF), ethambutol (EMB), and pyrazinamide (PZA). Due to the unique activity of PZA against persistent populations of bacilli, its inclusion in therapy has led to a reduction in treatment times from 9 to 6 months and has dramatically reduced disease relapse rates (2). Unfortunately, the increasing prevalence of drug-resistant TB infections compromises the viability of effective treatment regimens. In 2015, the estimated global incidence of PZA resistance was 16%, accounting for as many as 1.4 million cases (3). At least 70% of clinical PZA resistance can be attributed to loss-of-function mutations in *pncA*, which encodes an amidase that is essential for conversion of the drug to its active form, pyrazinoic acid (POA) (4). With the expansion of deep-sequencing-based approaches for characterization of molecular drug resistance mechanisms, there have been recent reports of PZA-resistant M. tuberculosis clinical isolates that carry wild-type *pncA* and harbor mutations in other genes with possible roles in resistance (5–7). An improved understanding of the molecular mechanisms that govern drug susceptibility and resistance will be crucial to advance genome-based molecular drug susceptibility testing and meet the challenge of ongoing trends in antimicrobial drug resistance.

Unlike other antimicrobial agents, PZA is active against M. tuberculosis only under specific environmental conditions *in vitro* and *in vivo*. Under standard culture conditions, PZA shows no growth-inhibitory activity, whereas, exposure of bacilli to low pH is known to drive PZA susceptibility (8). This observation is consistent with the finding that PZA shows no activity against M. tuberculosis within resting macrophages yet is bactericidal against bacilli within activated macrophages that undergo greater phagosomal acidification (9). Moreover, variable antitubercular PZA activity is observed in C3HeB/FeJ mice that show heterogeneous tuberculous lesions (10). In these mice, PZA is efficacious in lesions showing acidic pH but is ineffective against bacilli in lesions showing circumneutral pH (10). Further, PZA shows sterilizing activity against M. tuberculosis in immunocompetent mice yet lacks efficacy in athymic nude mice, which are unable to mount a cell-mediated immune response (11). Together, these studies demonstrate a pivotal role for host immunity in establishing sufficiently acidic microenvironments that are essential for the sterilizing antitubercular action of PZA (12).

As a potential explanation for the pH-dependent action of PZA, it was proposed that POA might function as a proton ionophore (13). In this model, PZA passively diffuses across the mycobacterial cell envelope and is then converted to POA anion by PncA. POA is then excluded from the cytoplasm by an unidentified efflux mechanism. A small proportion of POA (pK_a_, 2.9) becomes protonated upon exposure to an acidic environment and reenters the cell by passive diffusion. Protons then dissociate from incoming molecules of POA, and the cycle continues, leading to dissipation of proton motive force and acidification of the cytoplasm. In support of this model, intrabacterial acidification and dissipation of the proton motive force have been observed following 2 days of exposure of bacilli to PZA at pH 4.5 (14). However, it has not been demonstrated whether these events are due to POA acting as a protonophore or are the consequence of disruption of some other cellular process associated with maintenance of membrane potential. Importantly, proton motive force and intracellular pH are not measurably impacted by exposure of the bacilli to PZA at pH 5.8, which is typically used for PZA susceptibility testing (15). Further, pH-independent PZA susceptibility can be achieved through overexpression of PncA (15) or substitution of PZA with POA (15, 16). Thus, proton shuttling does not appear to be the basis for the pH-dependent action of PZA.

In addition to proton shuttling, several other modes of action for PZA have been proposed. These models include roles for POA in the inhibition of fatty acid synthesis, *trans*-translation, guanosine pentaphosphate synthetase/polyribonucleotide nucleotidyltransferase, quinolinic acid phosphoribosyltransferase, and coenzyme A (CoA) biosynthesis (17). While these models are not mutually exclusive, direct inhibition of fatty acid biosynthesis and *trans-*translation by POA have been challenged by subsequent studies (18, 19). Multiple recent reports confirm a connection between PZA action and impairment of CoA synthesis in M. tuberculosis. Mutations in *panD*, encoding the first enzyme of the CoA biosynthesis pathway, have been shown to confer PZA and POA resistance (20–22). Further, supplementation with intermediates of the CoA biosynthetic pathway was found to antagonize PZA and POA action (20, 22, 23). Moreover, POA exposure has been shown to significantly decrease intracellular levels of CoA in Mycobacterium bovis strain BCG (22, 24). Based on ligand interaction and cocrystallography studies, it has been demonstrated that POA binds PanD and promotes its destabilization (25–27). However, an M. tuberculosis strain with *panD* deleted shows measurable susceptibility to PZA (23), indicating that PanD is not the only target of POA. Despite these recent advancements in the understanding of PZA mechanism of action, their connection to conditional PZA susceptibility has yet to be determined.

In this study, we used the genome-wide deep-sequencing-based approach, transposon sequencing (Tn-seq), to comprehensively interrogate which cellular pathways are associated with PZA susceptibility. Genetic associations were identified across various cellular processes, many of which have not been previously linked with PZA or POA resistance. Many of these functions play key roles in central metabolism and the cell envelope stress response. Further, we demonstrate that activation of this response through the extracytoplasmic function sigma factor E (SigE) is central to conditional PZA susceptibility and can be manipulated by exposing the bacilli to antibiotics that target peptidoglycan synthesis. These observations establish a paradigm shift in our understanding of the action of this important drug through defining the regulatory mechanism that underlies conditional PZA susceptibility.

## RESULTS

### Genome-wide analysis of molecular mechanisms for pyrazinamide resistance.

Mutagenesis with the *himar1* transposon (28) was used to investigate the genetic basis for mycobacterial PZA susceptibility. To avoid a preponderance of insertion mutations in *pncA* and circumvent the need for acidification of the growth medium, strains were selected for resistance to POA at pH 6.6. Similar conditions were recently used for the identification of spontaneous POA resistance mutations in *panD* and *clpC1* in M. tuberculosis and M. bovis BCG (22, 29). It is important to note that while POA shows inhibitory activity against M. tuberculosis at circumneutral pH, POA susceptibility is enhanced by exposure to low pH. Since we utilize the mycobacteriophage phAE180 (30) for delivery of *himar1*, and susceptibility to PZA and POA can be modulated by specific extracellular stimuli, the impact of phage infection on POA susceptibility was determined. On 7H9 agar medium, the MIC of POA for M. tuberculosis H37Rv was 200 μg mL^−1^ at pH 6.6 and 50 μg mL^−1^ at pH 5.8 (Table S1), consistent with previous reports (16, 31). Interestingly, when bacilli were infected with phAE180 and plated on solid medium (pH 6.6) containing kanamycin for selection of *himar1* transposon insertion, the POA MIC was 50 μg mL^−1^ (Table S1). Thus, like exposure to low pH, phage infection also enhances susceptibility of M. tuberculosis to this drug. Enhancement of POA susceptibility was also observed for M. bovis BCG following infection with phAE180 (Table S1), demonstrating that phage-mediated potentiation of mycobacterial POA susceptibility is not strain specific.

10.1128/mBio.00439-21.1 TABLE S1 POA susceptibility of M. tuberculosis and M. bovis is enhanced by infection with mycobacteriophage phAE180, similar to exposure to low pH. Download Table S1, DOCX file, 0.06 MB. Copyright © 2022 Thiede et al. 2022 Thiede et al. https://creativecommons.org/licenses/by/4.0/ This content is distributed under the terms of the Creative Commons Attribution 4.0 International license.

Spontaneous resistance to POA at circumneutral pH has been reported to occur at a frequency of 10^−5^ (22). When approximately 2 × 10^5^ independent M. tuberculosis H37Rv transposon insertion mutants were plated on 50 μg mL^−1^ POA at pH 6.6, 2 × 10^3^ colonies emerged, indicating that ∼1% of insertions were associated with POA resistance. Fourteen independent isolates were chosen to assess POA and PZA resistance and to determine the corresponding chromosomal transposon insertion sites. Of these isolates, 10 had unique insertions and 4 appeared to be siblings. Consistent with other recent studies (20–22, 32–34), insertions were identified in the carboxy-terminal coding region of *panD* and in the promoter region of *clpC1* (Table S2). Eight additional unique insertions were identified within seven other genes (Table S2), four of which we recently associated with PZA or POA resistance (35). These strains showed 2- to 16-fold resistance to POA at pH 6.6 (MIC, 400 to 3,200 μg mL^−1^), whereas PZA resistance was typically only 2-fold at pH 5.8 (MIC, 100 μg mL^−1^) relative to the parental strain (Table S2), as was previously described for *panD* and *clpC1* mutant strains (32). It is noteworthy that despite ongoing debate over the breakpoint concentration for PZA, resistance of M. tuberculosis clinical isolates to >50 μg mL^−1^ is associated with poor sputum conversion rates (36). For all mutant strains that were tested, INH susceptibility was indistinguishable from that of the parental strain (Table S2), indicating that resistance was specific to PZA and POA.

10.1128/mBio.00439-21.2 TABLE S2 Genotypic and phenotypic characterization of M. tuberculosis H37Rv pyrazinamide-resistant transposon mutants. Download Table S2, DOCX file, 0.02 MB. Copyright © 2022 Thiede et al. 2022 Thiede et al. https://creativecommons.org/licenses/by/4.0/ This content is distributed under the terms of the Creative Commons Attribution 4.0 International license.

**FIG 1:**
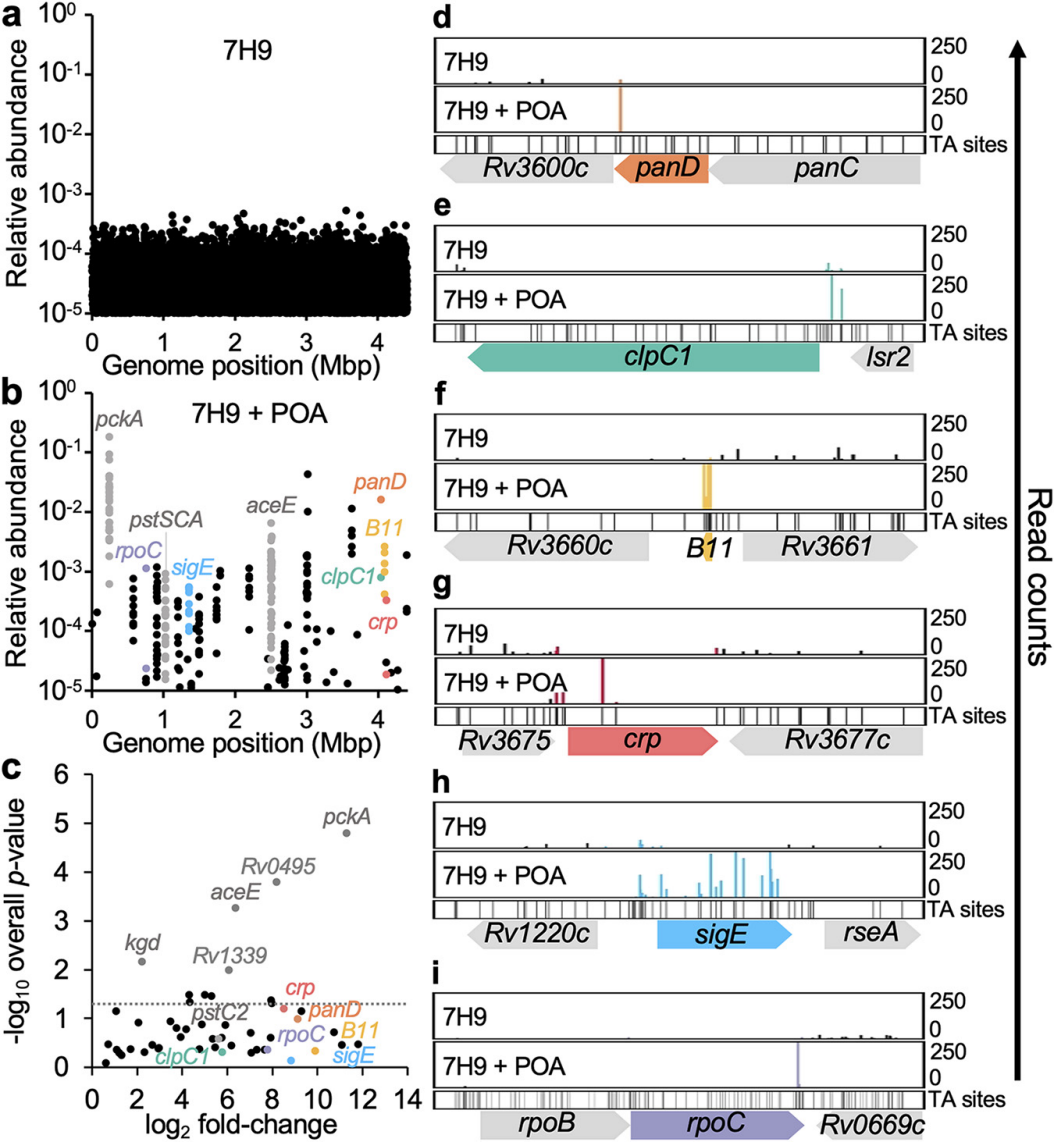
Genes associated with M. tuberculosis PZA susceptibility by Tn-seq. Libraries of 2 × 10^5^ independent M. tuberculosis H37Rv *himar1* insertion mutants (4-fold saturation) were plated on 7H9 agar without (a) or with (b) POA. Genomic DNA was extracted, processed, and sequenced as described by Minato et al. (37). (a and b) Mean abundance relative to total TA insertion read counts from two independent replicates. (c) Log_2_ fold changes in mean relative abundance from panel b compared to panel a by gene and respective −log_10_ *P* value (dotted line at *P = *0.05). (d to i) Read count comparisons for transposon insertions in *panD* (d), P*_clpC1_* (e), *B11* (f), *crp* (g), *sigE* (h), and *rpoC* (i).

To comprehensively identify genes associated with POA susceptibility, M. tuberculosis H37Rv was transposon mutagenized as described above, and an equal volume of mutagenized cells was plated on 7H9 medium without or with 50 μg mL^−1^ POA at pH 6.6, performed in biological duplicate. In the absence of POA, approximately 2 × 10^5^ colonies were recovered from each mutagenesis. Similar to that described above, selection with POA yielded approximately 2 × 10^3^ POA-resistant colonies from each mutagenesis. To identify transposon insertion sites, colonies were collected from each condition, genomic DNA was extracted, transposon-adjacent regions were enriched by PCR, and deep sequencing was performed (37). Sequencing of non-POA-selected colonies yielded 1.1 and 0.72 million high-quality reads that mapped to 46,901 and 34,635 unique TA sites, respectively (Fig. 1a; Data Set S1). Sequencing of POA-selected colonies resulted in 0.97 and 0.48 million high-quality reads that mapped to 9,903 and 1,058 unique TA sites, respectively (Data Set S1). Insertion sites that were present at an abundance of ≥5 read counts in both POA-selected data sets were analyzed further. These insertions constituted >93% of total reads and mapped to 275 TA sites within 58 genes and intergenic regions (Fig. 1b; Table S3). The remaining reads were low abundance (<5 read counts, 1% of reads) and/or observed only in one replicate (6% of reads) and were excluded from further analysis. Fold enrichment for insertions in highly represented loci was determined by comparing the mean relative read abundance to that from the no-POA condition (Fig. 1c; Table S3). Eleven genes showed a level of enrichment between 2- and 1,000-fold in the presence of POA and achieved a threshold of significance of <0.05 (Fig. 1c; Table S3).

10.1128/mBio.00439-21.3 TABLE S3 Fold change in relative abundance of *himar1* insertions in M. tuberculosis loci following selection on medium containing POA. Download Table S3, DOCX file, 0.1 MB. Copyright © 2022 Thiede et al. 2022 Thiede et al. https://creativecommons.org/licenses/by/4.0/ This content is distributed under the terms of the Creative Commons Attribution 4.0 International license.

10.1128/mBio.00439-21.5 DATA SET S1 Tn-seq read count data and statistical analysis for POA-resistant *himar1* insertion mutant strains. Download Data Set S1, XLSX file, 6.3 MB. Copyright © 2022 Thiede et al. 2022 Thiede et al. https://creativecommons.org/licenses/by/4.0/ This content is distributed under the terms of the Creative Commons Attribution 4.0 International license.

The majority of highly represented insertions were identified in loci that are functionally associated with intermediary metabolism and respiration, cell wall and cell processes, information pathways, stable RNAs, virulence-detoxification-adaptation, and conserved hypothetical proteins (Table 1). As anticipated, highly abundant insertions were identified in the 3′ end of *panD* (Fig. 1b to d) and promoter of *clpC1* (Fig. 1b, c, and e), yet these insertions fell below the significance threshold due to a limited number of available TA sites. Numerous insertions in genes for the persistence-associated high-affinity phosphate transport system (38, 39) were observed (Fig. 1b and c; Fig. S1a), consistent with reports of point mutations in *pstC2* in PZA-resistant laboratory and clinical isolates (6, 33). Further, highly abundant insertion sites also included eight of the 10 genes that were identified in our primary analysis (described in Table S2).

**TABLE 1:** Loci of highly abundant transposon insertions associated with pyrazinoic acid resistance in M. tuberculosis

Gene name(s)	Functional category^*a*^	Functional role^*a*^	No. of unique insertions^*b*^	Mean relative abundance per 100 reads^*c*^
*pckA*	Intermediary metabolism and respiration	Gluconeogenic phosphoenolpyruvate carboxykinase	31 (0.84)	73.0
*aceE*	Intermediary metabolism and respiration	E1 subunit of pyruvate dehydrogenase	49 (0.79)	5.3
*Rv3256c*	Cell wall and cell processes	Membrane protein associated with mannosylation of envelope lipids	5 (0.5)	2.5
*Rv2690c*	Cell wall and cell processes	Probable integral membrane protein	15 (0.41)	1.4
*phoT*, *pstS2*, *pstC2*, *pstA1*	Cell wall and cell processes	High affinity phosphate transport system	33 (0.45)	1.0
*subI*, *cysT*, *cysW*, *cysA1*	Cell wall and cell processes	Sulfate transport system	17 (0.27)	0.053
*sigE*	Information pathways	Extracytoplasmic function sigma factor	9 (0.36)	0.29
*ncRv13660c*	Small stable RNA	Small RNA that regulates *panD* mRNA stability	5 (0.83)	0.73
*Rv1957*	Virulence, detoxification and adaptation	Chaperone involved in modulation of HigAB toxin-antitoxin activity	7 (0.54)	0.42
*Rv2706c*	Conserved hypothetical protein	Unknown function	2 (1.0)	5.3

a Functional categories and functional roles are based on descriptions in the Mycobrowser portal at https://mycobrowser.epfl.ch.

b Number of unique insertions that were observed at ≥5 reads in both biological replicates; numbers in parentheses are fractions of total TA sites per gene(s) showing ≥5 reads.

c Mean relative abundance of reads for insertions observed at ≥5 reads in both biological replicates.

10.1128/mBio.00439-21.4 FIG S1 Genes associated with M. tuberculosis PZA susceptibility by Tn-seq. Libraries of 2 × 10^5^ independent *himar1* mutants (4-fold saturation) were plated on 7H9 agar without (top panels; 7H9) or with (middle panels; 7H9 + POA) POA. Genomic DNA was extracted, processed, and sequenced as described by Minato et al. (37). Bottom panels show all TA dinucleotides of the region that is illustrated. Mean read count comparisons from two independent replicates are shown for *pstS3C2A1* (a), *pckA* (b), *aceE* (c), and *subI*-*cysTWA1* (d). Download FIG S1, PDF file, 0.6 MB. Copyright © 2022 Thiede et al. 2022 Thiede et al. https://creativecommons.org/licenses/by/4.0/ This content is distributed under the terms of the Creative Commons Attribution 4.0 International license.

More than 80% of highly represented insertions in this analysis were located in genes involved in central carbon metabolism. Insertions throughout *pckA* (encoding phosphoenolpyruvate carboxykinase, the first enzyme of gluconeogenesis) constituted the vast majority (73%) of reads (Fig. 1b and c; Fig. S1b). The second most abundant set of insertions were found throughout *aceE* (encoding the E1 subunit of pyruvate dehydrogenase) and represented 5.3% of all reads (Fig. 1b and c; Fig. S1c). To confirm that loss-of-function mutations in *pckA* and *aceE* can confer POA resistance, these genes were deleted from H37Rv using the recombineering-based approach ORBIT (40). Both strains were found to be at least 4-fold more resistant to POA (MIC, ≥800 μg mL^−1^) than H37Rv (MIC, 200 μg mL^−1^). Less abundant insertions were reproducibly observed in the promoter region for *dlaT* (encoding the E2 subunit of pyruvate dehydrogenase) and within *icd2* (encoding isocitrate dehydrogenase) and *kgd* (encoding alpha-ketoglutarate decarboxylase) (Fig. 1c; Table 1; Table S3) indicating an important association between POA action and central carbon metabolism.

Growth at low pH is known to result in extensive metabolic remodeling that is dependent upon PckA (41), which may be an important event linked to PZA potentiation. Thus, it is likely that these mutations mitigate the deleterious impact of POA on CoA biosynthesis through increasing availability of oxaloacetate for production of l-aspartate, the precursor of β-alanine. Consistent with this model, we found abundant insertions in *B11* (*ncRv13660c*) (Fig. 1b, c, and f), a *trans*-encoded small RNA that regulates CoA synthesis through duplex formation with *panD* mRNA (42). Insertions were also observed in *crp* (*Rv3676*) (Fig. 1b, c, and g) encoding the transcriptional regulator of *B11* (43). In addition, insertions were also identified in genes for the sulfate transport system encoded by the *subI-cysTWA1* operon (Table 1; Fig. S1d). In contrast to our findings with POA, this operon was recently described for its role in intrinsic tolerance to many other antitubercular drugs (44). Similarly, abundant insertions were found in *sigE* (Fig. 1b, c, and h), encoding an extracytoplasmic function sigma factor that has been implicated in broad drug tolerance of M. tuberculosis and response to cell envelope stress (45–47). Further, abundant insertions were observed in the carboxy-terminal domain of *rpoC* (Fig. 1b, c, and i), which mediates critical interactions between SigE and RNA polymerase (48). Together, these observations indicate contrasting roles for genes associated with POA susceptibility versus tolerance to other drugs that target actively replicating bacilli, consistent with the long-standing notion that PZA predominantly targets persister populations.

### Activation of the cell envelope stress response potentiates PZA susceptibility.

Based on the new findings described above and the established role of SigE in response to low pH (45), it is likely that acidic conditions drive PZA and POA susceptibility through activation of the cell envelope stress response. To confirm a role for this response as a driver of PZA and POA action, susceptibility of a strain with a deletion of *sigE* (Δ*sigE*) was tested in liquid medium. For wild-type M. tuberculosis H37Rv, the MIC of PZA was 50 μg mL^−1^ and that of POA was 200 μg mL^−1^ (Table 2). In contrast, the MIC of PZA was 400 μg mL^−1^ and that of POA was ≥800 μg mL^−1^ for the M. tuberculosis Δ*sigE* strain (Table 2), confirming SigE as a critical driver of susceptibility. Expression of *sigE* from an integrating vector was sufficient to restore susceptibility of the Δ*sigE* strain to both PZA and POA (Table 2). INH susceptibility was indistinguishable for all strains, indicating that the association between *sigE* and drug resistance is specific to PZA and POA (Table 2). Indeed, in contrast to the role for SigE in conditional susceptibility to PZA, recent work by Pisu and colleagues demonstrates that the SigE response is important for mediating tolerance of M. tuberculosis to numerous other antimicrobial agents (46).

**TABLE 2:** The SigE response plays a central role in PZA and POA susceptibility of M. tuberculosis

M. tuberculosis strain	Relevant characteristic	MIC (μg mL^−1^)^*a*^		
		POA	PZA	INH
H37Rv	Parental strain	200	50	0.0625
H37Rv Δ*sigE*	H37Rv with deletion of sigma factor E gene	≥800	400	0.0625
H37Rv Δ*sigE* pMV306-*sigE*	Δ*sigE* strain expressing *sigE* in *trans*	400	50-100	0.0625

a MIC is defined as the minimum concentration required to inhibit 90% of growth relative to the no-drug control after 14 days (for POA and PZA) or 7 days (for INH) of incubation at 37ºC. POA and INH exposure was performed at pH 6.6; PZA exposure was performed at pH 5.8.

**FIG 2:**
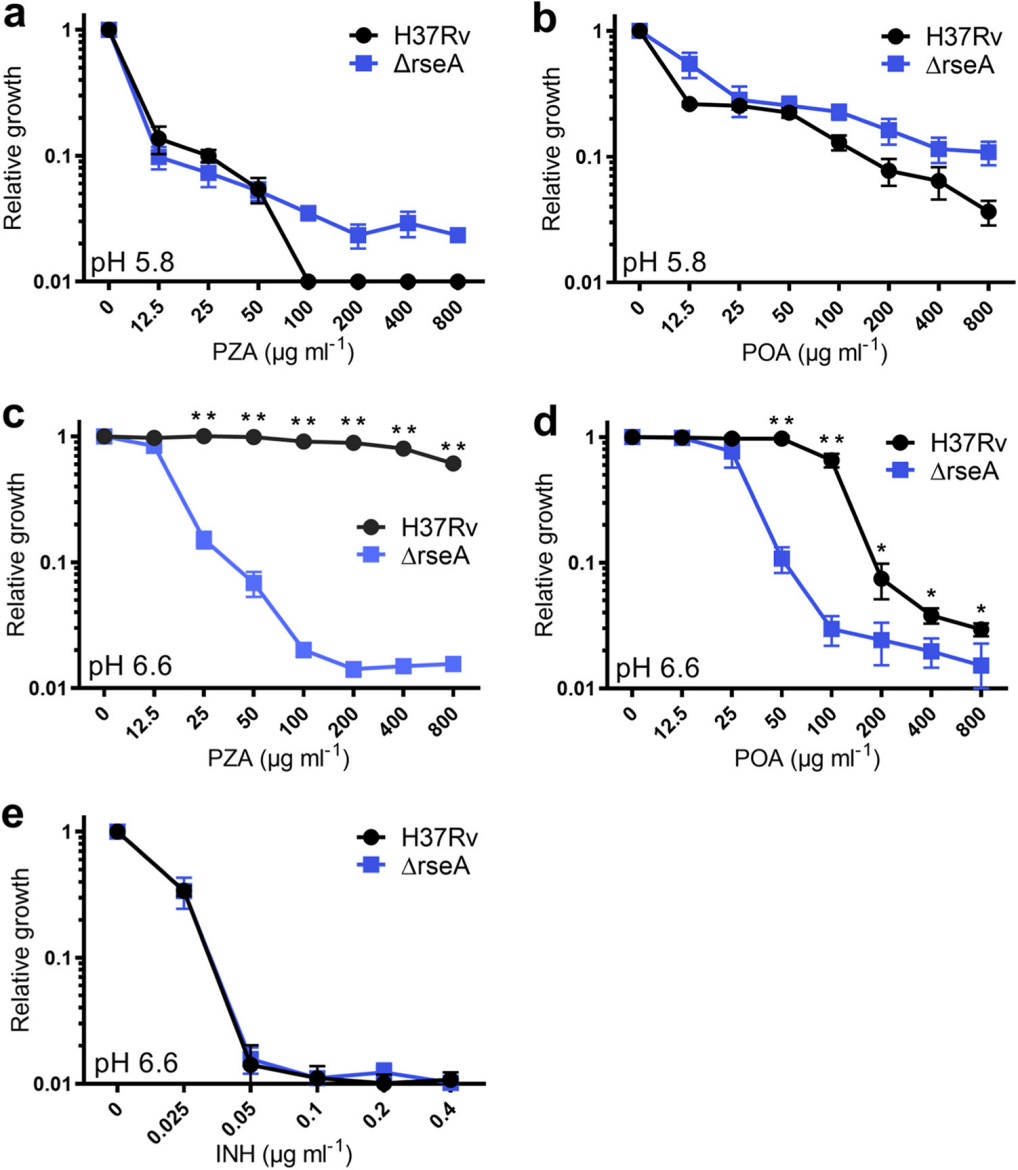
Deletion of *rseA* in M. tuberculosis confers constitutive susceptibility to PZA. M. tuberculosis H37Rv (wild type, black circles) and the Δ*rseA* strain (blue squares) were grown in 7H9 at pH 5.8 (a and b) or pH 6.6 (c to e) and exposed to PZA (a and c) or POA (b and d) for 14 days or INH for 7 days (e). OD_600_ was measured, and relative growth was determined by dividing by the value of the no-drug control. Data are means and standard deviations (SD) for 3 biological replicates. *, *P* < 0.05, and **, *P* < 0.0002, by 2-tailed Student's *t* test.

Under nonstress conditions, the cell envelope stress response is muted through sequestration of SigE by the anti-sigma factor RseA (49). Upon sensing of cell envelope stress, RseA is phosphorylated by the essential serine-threonine kinase PknB and is subsequently degraded by the ClpC1P1P2 protease, resulting in release of SigE and thereby promoting expression of its regulon (50). To further evaluate the role of the SigE response in susceptibility of M. tuberculosis to PZA and POA, a strain with a deletion of *rseA* (Δ*rseA*) was assessed for PZA and POA susceptibility under acidic (pH 5.8) and circumneutral (pH 6.6) incubation conditions. As anticipated, under acidic conditions, the Δ*rseA* strain showed a level of PZA and POA susceptibility that was indistinguishable from that of the wild-type control (Fig. 2a and b). Also, as has been previously described, when wild-type H37Rv was exposed to concentrations up to 800 μg mL^−1^ PZA at circumneutral pH, growth was not notably impaired (Fig. 2c). In striking contrast, when the Δ*rseA* strain was exposed to PZA under circumneutral conditions (Fig. 2c), it showed a level of susceptibility comparable to that which was observed under acidic conditions (Fig. 2a). Similarly, susceptibility of the Δ*rseA* strain to POA was equivalent under circumneutral (Fig. 2d) and acidic (Fig. 2b) conditions. Importantly, INH susceptibility was unchanged (Fig. 2e), indicating that the potentiation effect through constitutive activation of the SigE response is specific to PZA and POA. Taken together, these results demonstrate that activation of the SigE cell envelope stress response is both necessary and sufficient for driving PZA and POA susceptibility in M. tuberculosis.

**FIG 3:**
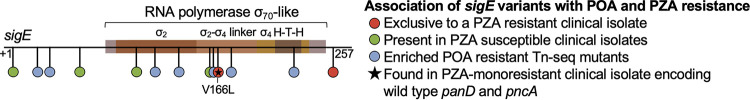
Distribution of transposon insertions and natural sequence polymorphisms observed in clinical isolates in SigE functional domains. Location of Tn-seq insertions enriched under POA pressure and nonsynonymous single nucleotide polymorphisms (SNPs) observed across PZA-monoresistant clinical isolates with respect to SigE domain architecture imported from InterPro and curated from recent literature (51). Locations of all missense mutations present in at least one isolate and all TA insertion sites with greater relative abundance in POA-containing media relative to the 7H9 control are depicted.

Next, we evaluated whether there were any signatures of enrichment for *sigE* mutations in PZA-resistant clinical isolates. Considering that most clinical PZA resistance is mediated by *pncA* mutations and an intact SigE regulon is essential for tolerating other antitubercular therapeutics (46), we reasoned that any enrichment for *sigE* mutants would be exceedingly rare and appear more frequently—if not exclusively—in *pncA*_WT_ PZA-monoresistant isolates. Accordingly, we contrasted the prevalence of *sigE* mutations in a large global set of clinical isolates (*n *= 1,215) to a recently curated set of PZA-monoresistant isolates (*n *= 18) in which *pncA_WT_* isolates were markedly overrepresented (34), including three that are both *pncA*_WT_ and *panD*_WT_. Among all isolates, six nonsynonymous *sigE* mutations were observed. Among these, *sigE*_V166L_ was most tenable for conferring an advantageous functional effect. The V166L substitution falls within the SigE σ_2_/σ_4_ linker (Fig. 3), which was recently shown to interface with template single-stranded DNA in the active center cleft of RNA polymerase, and influences open-complex formation, abortive production, and promoter escape during transcription initiation (51). A mutation in this linker domain could affect how SigE associates with promoters of its regulon, ostensibly altering the conditionality or specificity of its transcription-activating action without uniformly deleterious effects. The single isolate harboring this mutation was one of only three PZA-monoresistant, PncA_WT_, and PanD_WT_ isolates. The presence of this mutation in a lone isolate monoresistant to PZA and lacking the two most established resistance-conferring clinical mutations suggests a potential role in PZA resistance. This finding is consistent with the idea that the SigE dependence of PZA susceptibility we have characterized *in vitro* might also operate within the context of human infection.

### Peptidoglycan-targeting agents strongly potentiate PZA antitubercular action.

**FIG 4:**
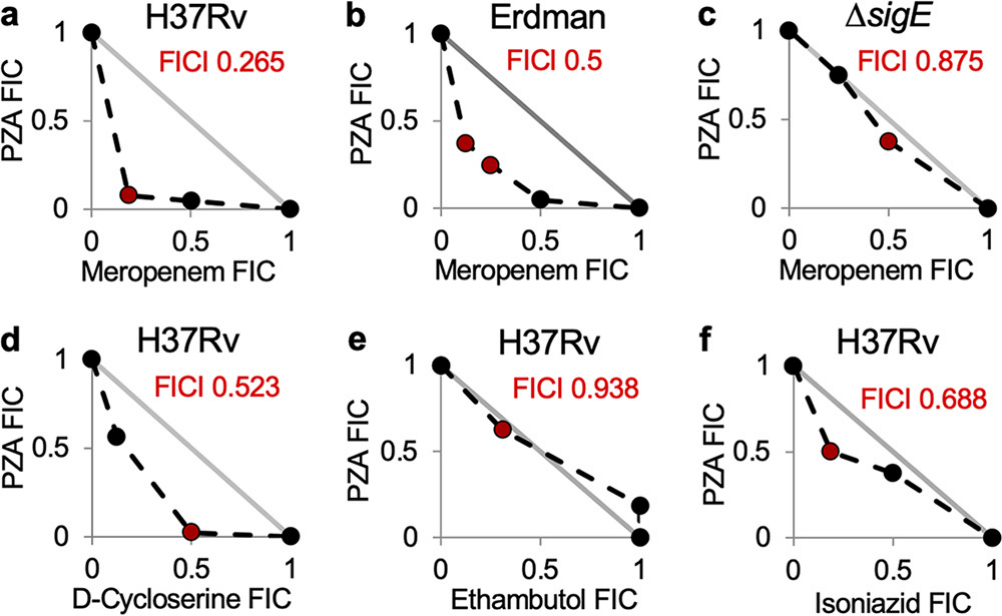
Peptidoglycan synthesis inhibitors potentiate antitubercular activity of pyrazinamide. M. tuberculosis H37Rv (a and d to f), Erdman (b) and H37Rv Δ*sigE* (c) were grown in 7H9 at pH 6.6 with various concentrations of PZA and either meropenem/clavulanate (a to c), d-cycloserine (d), ethambutol (e), or isoniazid (f) in checkerboard format. OD_600_ was determined after 7 days of incubation. Plots were generated based on the average fractional inhibitory concentration (FIC) calculated from two biological replicates. The lowest FIC index (FICI) values are indicated in red. Line of additivity is shown in gray (FICI of 1).

Based on the observation that the SigE response governs PZA conditional susceptibility, we reasoned that specific activation of this response through exposure to cell envelope damaging agents should lead to potentiation of PZA activity against M. tuberculosis. To assess the impact of differing types of cell envelope stress on PZA activity, compounds disrupting synthesis of various layers within the mycobacterial cell envelope were evaluated for fractional inhibitory concentration index (FICI) values using checkerboard assays in standard 7H9 medium at pH 6.6. Meropenem and d-cycloserine were chosen to target peptidoglycan biosynthesis. Meropenem, a β-lactam, irreversibly inhibits penicillin-binding proteins (PBPs), thereby preventing cross-linking of the peptidoglycan side chains. The β-lactamase inhibitor clavulanate was included to inhibit the M. tuberculosis β-lactamase and improve stability of meropenem (52). Combining meropenem-clavulanate with PZA in a checkerboard assay yielded synergistic FICI values of 0.265 and 0.5 for M. tuberculosis H37Rv (Fig. 4a) and M. tuberculosis Erdman (Fig. 4b), respectively. Further, the minimum FICI value achieved for H37Rv Δ*sigE* was 0.875, indicating no drug interaction (Fig. 4c). Thus, the synergistic activity between meropenem and PZA is dependent upon the SigE response.

Based on the observed synergy between meropenem and PZA, we assessed whether the mechanism of peptidoglycan damage impacted potentiation of PZA action. d-Cycloserine is an amino acid analogue that targets l-alanine racemase and d-alanyl-alanine synthetase, preventing synthesis of new peptidoglycan, as opposed to blocking PBP cross-linking. Checkerboard assays for d-cycloserine and PZA yielded a minimum FICI value of 0.523 (Fig. 4d), indicating an additive effect between these agents, in contrast to the synergy observed between PZA and meropenem.

Next, we investigated whether drugs targeting other mycobacterial envelope layers were also capable of enhancing susceptibility to PZA. EMB was chosen to target the arabinosyl transferases that are essential for synthesis of the arabinogalactan layer of the mycobacterial envelope (53). Similar to that which was observed with d-cycloserine, EMB exposure also did not potentiate PZA activity (Fig. 4e). The lowest FICI value achieved was 0.938, indicating an additive effect. INH was chosen to target the mycolic acid layer. INH inhibits InhA, the enoyl acyl carrier protein (ACP) reductase, preventing mycolic acid synthesis (54). Similar to EMB, cotreatment with INH showed an additive effect on PZA susceptibility (Fig. 4f). These observations are in agreement with previous observations that INH and PZA do not show substantial interaction in culture-based assays (55).

## DISCUSSION

**FIG 5:**
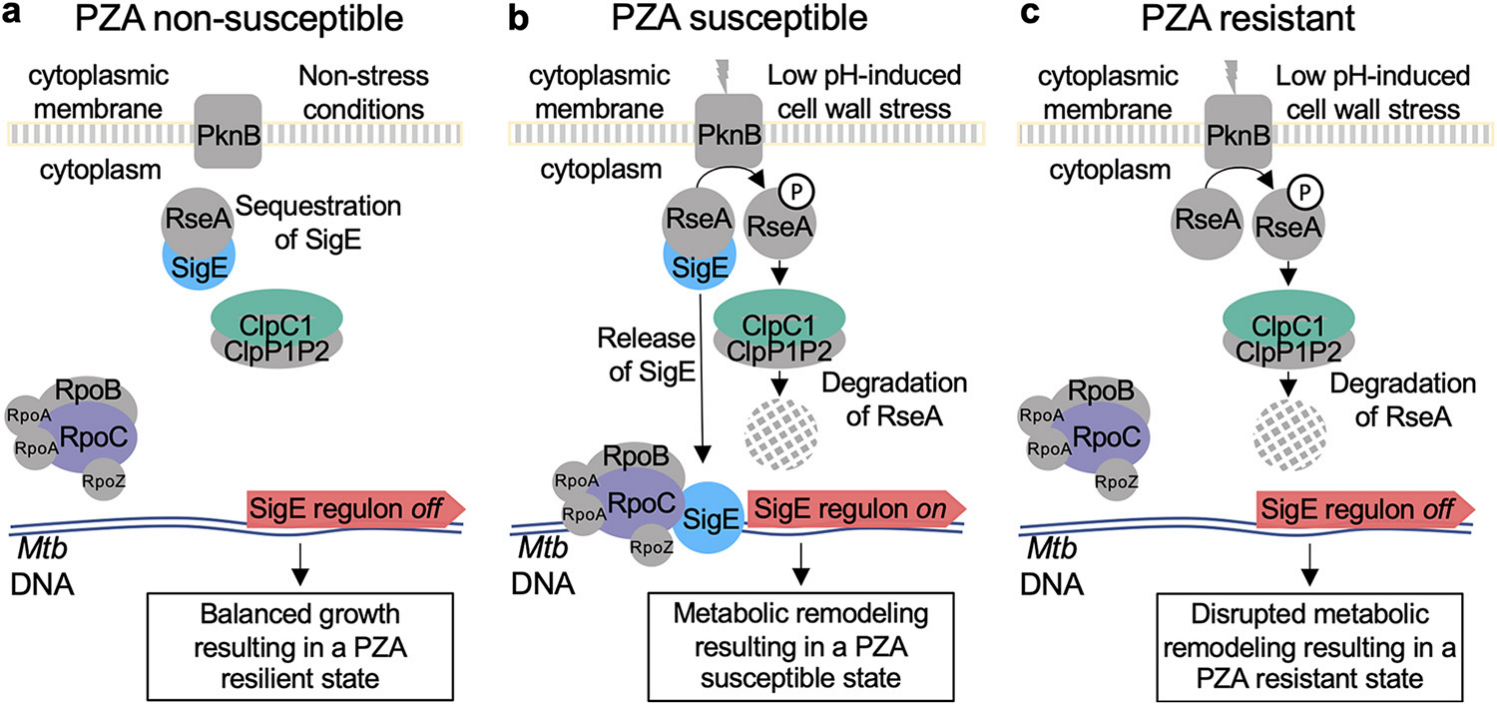
Model for low pH-mediated potentiation of PZA susceptibility. (a) Under conditions of balanced growth, M. tuberculosis is in a nonstressed state, resulting in PZA resilience. (b) Under conditions of low pH or other cell wall stress-inducing conditions, signaling through PknB mediates phosphorylation of RseA, which is subsequently degraded by ClpC1P1P2, liberating SigE. Expression of the SigE regulon results in metabolic remodeling that poises bacilli in a PZA-susceptible state. (c) Failure to activate the SigE response prevents cell wall stress-driven metabolic remodeling, resulting in PZA resistance.

Despite several decades of study and clinical use, the mechanistic basis for conditional susceptibility of M. tuberculosis to PZA remained elusive. In this study, we utilized a genome-scale approach to reveal cellular responses that drive PZA susceptibility and are orchestrated through activation of the cell envelope stress response that is governed by the alternate sigma factor SigE (Fig. 5). Indeed, we demonstrate that constitutive activation of this stress response through deletion of the anti-sigma factor gene *rseA* renders M. tuberculosis intrinsically susceptible to PZA, whereas blocking this response leads to resistance. Resistance was also observed when activation of the SigE response was impaired through disruption of the *clpC1* locus, which plays a central role in degradation of RseA. Moreover, we demonstrated that activation of the cell envelope stress response through cotreatment with peptidoglycan-damaging agents leads to potent potentiation of PZA and is SigE dependent. These observations offer a stark contrast to the relationship between the SigE response and other antimicrobial agents, where this response has been shown to promote tolerance to numerous other antitubercular drugs, such as INH, EMB, RIF, and streptomycin (46). The precise role that SigE has in promoting survival during stress treatment has not been fully elucidated, but recent studies have suggested that the regulon may play a role in maintenance of M. tuberculosis dormancy (56). SigE-dependent maintenance of dormancy may be accomplished through modulation of central metabolism (47) or through the MprAB-SigE driven regulation of a Psp-like system that maintains M. tuberculosis cell envelope integrity (57). These connections between SigE and dormancy that lead to increased tolerance to multiple antibiotics may be a double-edged sword in the case of PZA treatment, offering a potential explanation for PZA efficacy against M. tuberculosis independent of growth state.

One counterintuitive finding in our study is the observation that disruption of ClpC1 and SigE conferred measurable resistance to POA in the absence of external cellular stress. This finding is not unprecedented given the well-established role of PknB in activation of the SigE response through phosphorylation of RseA (50). PknB is an essential transmembrane serine/threonine protein kinase that contains an extracellular peptidoglycan-sensing domain (58). Through sensing peptidoglycan fragments present at the septum, PknB plays a direct role in determining cell shape and controlling cell division (59–61). It stands to reason that the SigE response might be transiently activated during cell division, in addition to its well-described role in stress response. Activation of the SigE regulon during cell division could explain M. tuberculosis susceptibility to POA under nonstress conditions, as well as cases where pH has been uncoupled from PZA and POA susceptibility (15). In support of this notion, a recent study by Bandekar et al. (62) which examined temporal expression of all transcripts in a synchronized population of M. tuberculosis demonstrated periodic induction of *sigE* expression midway through the mycobacterial cell division cycle. Further studies are essential to understand the consequence of this periodicity on conditional drug susceptibility.

Currently, the biochemical event linking activation of the SigE regulon to PZA susceptibility is unclear. Many of the highly abundant hits in our screen were mapped to genes capable of impacting cellular CoA levels. These observations are consistent with previous reports linking mutations in CoA-biosynthetic genes as well as supplementation with CoA precursor molecules to PZA resistance (20, 22, 23). Gopal and colleagues recently showed that intracellular CoA pools play a major role in determining PZA and POA susceptibility (22). Phosphoenolpyruvate carboxykinase, α-ketoglutarate dehydrogenase and pyruvate dehydrogenase play key roles in central carbon metabolism where CoA is used at various steps to move carbon into, through, and out of the tricarboxylic acid (TCA) cycle. Changes in the proteome during stress response conditions are expected to impact CoA-dependent metabolism. Exposure to acidic pH leads to specific metabolic remodeling that provides a fitness advantage (41). The SigE response likely orchestrates this adaptation through major transcriptional remodeling (46, 63). These data, taken with the previous finding that POA treatment decreases intracellular abundance of CoA (22), suggest that perturbations in CoA levels influence M. tuberculosis susceptibility to PZA and POA. We propose that cell envelope stress leads to an induction of the SigE regulon, which in turn modulates cellular CoA levels. Once CoA levels have been reduced, treatment with PZA or POA further depletes CoA, leading to growth inhibition through metabolic dysfunction (Fig. 5). Further support for this model is the observation that supplementation with exogenous CoA precursors can antagonize PZA and POA action (20, 22, 23). These observations also suggest that CoA-dependent metabolism may determine the impact of PZA and POA treatment. Under conditions that do not induce the SigE response, PZA and POA are needed at relatively high concentrations to exert a strong enough effect on intracellular CoA and impact growth. However, under conditions that trigger the SigE response, intracellular CoA levels are more vulnerable to perturbations by PZA or POA.

The ability to disconnect PZA susceptibility from pH allows isolation of informative resistant mutants that have been missed by previous screens and selections. There may be an *in vivo* fitness cost providing a strong counterselection against certain mutations during infection. For example, both *panD* and *aceE* have been characterized as essential genes for growth in a mammalian host (64, 65). These findings offer new insight into characterization of clinically resistant strains that do not have mutations in loci that are commonly associated with PZA resistance, such as *pncA*. A recently sequenced PZA clinical isolate described by Maslov et al. harbored 15 nonsynonymous mutations in protein coding sequences (5), and it was concluded that further functional analysis would be needed to associate any of these mutations to the observed PZA resistance. In light of our findings, it is possible to correlate these putative resistance mutations with several highly abundant loci detected in our study, namely, *aceE*, *rpoC*, and *dlaT*, as candidates contributing to PZA resistance. Independently, each of these mutations is likely to confer a low level of PZA resistance. However, in combination, the effect of these mutations may be additive and culminate in a high level of resistance to PZA. Whole-genome-based polygenic surveys for loci associated with PZA resistance may offer a powerful predictive tool for rapid molecular PZA susceptibility testing.

This new understanding of conditional PZA susceptibility may be particularly impactful in the context of TB therapy in individuals with compromised immunity. During TB infection, bacilli are engulfed by dendritic cells and alveolar macrophages, where they reside within phagosomal compartments (66). Prior to activation by helper T cell signals, the bacilli are able to replicate within phagosomes despite conditions of mild acidity (pH 6.2 to 6.4) and exposure to subinhibitory levels of reactive oxygen intermediates (66). Following cell-mediated activation by proinflammatory cytokines, such as gamma interferon and tumor necrosis factor alpha, intracellular bacilli must adapt to more severe growth-inhibitory stressors, such as lowered pH (pH 4.5 to 5.4), nutrient limitation, and bombardment with high levels of reactive oxygen and reactive nitrogen intermediates (66). While T cell-mediated activation of macrophages is not usually sufficient to eliminate all bacilli from the host, it is essential for long-term containment in the form of a latent infection (66). Impairment of CD4^+^ T cell signals through prolonged antigen stimulation, genetic lesions in the gamma interferon signaling pathway, anti-tumor necrosis factor alpha therapy, and HIV coinfection are common mechanisms that drive progression to overt TB disease and other mycobacterial infections (67). Consistent with a role for host involvement in the sterilizing activity of PZA, it was recently demonstrated that PZA-mediated killing is impaired in athymic nude mice, which are unable to drive cell-mediated activation of monocytes due to a complete lack of T cells (11). Combined with our findings, this essential role for T cell responses for *in vivo* PZA action suggests that activation of host antimicrobial stressors is critical for driving PZA susceptibility of M. tuberculosis. As such, these observations indicate that PZA might have suboptimal activity in individuals with compromised cell-mediated immunity. Our findings open up potential avenues to improve PZA efficacy in the context of immune deficiency through the adjunctive use of carbapenems to drive the cell envelope stress response.

## MATERIALS AND METHODS

### Bacterial strains and growth conditions.

Middlebrook 7H9 medium (Difco) supplemented with 10% (vol/vol) oleic acid-albumin-dextrose-catalase (OADC, Difco), 0.2% (vol/vol) glycerol, and 0.05% (vol/vol) tyloxapol (Sigma) and 7H10 medium (Difco) supplemented with 10% (vol/vol) OADC and 0.2% (vol/vol) glycerol were used to cultivate M. tuberculosis H37Rv and Erdman and M. bovis BCG. When necessary, kanamycin (Thermo Scientific) and/or hygromycin (Corning) were added at 50 and 150 μg mL^−1^, respectively.

### Transposon mutagenesis and transposon sequencing.

M. tuberculosis H37Rv was mutagenized with the mariner *himar1* transposon using the temperature-sensitive mycobacteriophage phAE180^30^. Approximately 2 × 10^5^ independent transposon-mutagenized bacilli were spread on Middlebrook 7H9 medium supplemented with 10% (vol/vol) OADC and 0.2% (vol/vol) glycerol containing 1.5% agar (Difco) and 50 μg mL^−1^ kanamycin, without or with 50 μg mL^−1^ POA (Sigma), in 245-mm^2^ BioAssay dishes (Nunc). For analysis of individual POA-resistant strains, secondary plating was performed on supplemented 7H10 medium without or with 400 μg mL^−1^ POA.

For transposon sequencing, plates were incubated for 2 weeks, colonies were collected, and genomic DNA was extracted as previously described (37). Briefly, colonies were scraped into 10 mL of 7H9 complete medium, and the mycobacteria were pelleted and incubated at 80°C for 2 h. Cultures were pelleted, resuspended, and washed in 500 μL of 25 mM Tris (pH 7.9), 10 mM EDTA, and 50 mM glucose. The cells were resuspended in 450 μL of 25 mM Tris (pH 7.9), 10 mM EDTA, and 50 mM glucose with 50 μL of 10 mg mL^−1^ lysozyme. Samples were incubated at 37°C for 16 h. Next, 100 μL of 10% SDS and 50 μL of 10 mg mL^−1^ proteinase K were added, and the mixture was incubated at 55°C for 30 min. Two hundred microliters of 5 M NaCl and 160 μL of CTAB saline solution (0.7 M NaCl, 0.275 M hexadecyl-trimethylammonium bromide [CTAB]) were added, and the samples were incubated at 65°C for 10 min.

DNA was extracted using multiple chloroform-isoamyl alcohol treatments. The DNA was precipitated with isopropanol and washed with 70% ethanol prior to its resuspension in EB buffer (Qiagen). DNA fragmentation and Illumina P7 adapter (CAAGCAGAAGACGGCATACGAGAT) ligation were performed in the NeoPrep library prep system (Illumina). Transposon junctions were amplified by using a transposon-specific primer, Mariner_1R_TnSeq_noMm (TCGTCGGCAGCGTCAGATGTGTATAAGAGACAGCCGGGGACTTATCAGCCAACC), and primer P7 (CAAGCAGAAGACGGCATACGAGAT) with a HotStarTaq master mix kit (Qiagen). The following PCR conditions were used: 94°C for 3 min, 19 cycles of 94°C for 30 s, 65°C for 30 s, and 72°C for 30 s, and 72°C for 10 min. The *himar1*-enriched samples were diluted 1:50 and amplified by using a P5 indexing primer (AATGATACGGCGACCACCGAGATCTACAC[i5]TCGTCGGCAGCGTC; [i5] barcode sequence) and a P7 primer HotStarTaq master mix kit (Qiagen) to add unique barcodes and the necessary P5 and P7 flow cell adapter sites for Illumina sequencing. The following PCR conditions were used: 94°C for 3 min, 94°C for 30 s, 55°C for 30 s, and 72°C for 30 s.

Sequencing was performed on an Illumina MiSeq system by the University of Minnesota Genomics Center. Transposon and adapter sequences were trimmed from the 5′ end of sequencing reads using Cutadapt (68). We also discarded all the sequence reads that did not contain adapter sequence in the 5′ trimming process. After the 5′ trimming process, all the sequence reads begin with TA. Adapter sequences were trimmed and sequence reads that were shorter than 18 bp were discarded. The default error rate of 0.1 was used for all trimming processes. The trimmed sequence reads were mapped (allowing 1 bp mismatch) to the M. tuberculosis H37Rv genome (GenBank no. AL123456.3) using Bowtie (69). The genome-mapped sequence reads were printed in SAM format, and sequence reads per each TA dinucleotide site in the M. tuberculosis H37Rv genome were counted using SAMreader_TA script (70).

The relative abundance of read counts for each TA site was calculated relative to total read counts for the respective library. Fold change in relative abundance of each TA site was determined by dividing mean relative abundance from the POA data set by mean relative abundance from the 7H9 data set. *P* values comparing relative abundance of TA sites without and with POA were determined by using a two-tailed paired Student's *t* test. Mean fold change in abundance per locus was determined by calculating the mean fold change in relative abundance of each TA site for a given locus. Overall *P* value per locus was determined by using Fisher’s combined probability test for each TA site within a given locus.

### Determination of drug susceptibility.

Strains were cultured to mid-log phase and subsequently inoculated at an initial optical density at 600 nm (OD_600_) of 0.01 into supplemented 7H9 medium at the indicated pH. The MIC was defined as the minimum concentration of drug required to inhibit at least 90% of growth relative to the drug free controls and was determined by measuring OD_600_. Drug susceptibility testing for PZA and POA was carried out using media at pH 5.8 or pH 6.6 with 14 days of incubation at 37°C. INH MIC determinations were carried out in medium at pH 6.6 with 10 days of incubation at 37°C. POA agar MIC determinations were conducted using 7H10 medium at pH 6.6. M. tuberculosis strains were spot diluted onto plates containing various concentrations of POA. MIC was determined at day 14 by assessing colony growth.

### Determining mycobacteriophage-mediated potentiation of POA activity.

To determine the impact of mycobacteriophage transduction on POA susceptibility, M. tuberculosis and M. bovis strains were grown to an OD_600_ of 0.5. Cultures were pelleted by centrifugation and washed twice in an equal volume MP buffer (50 mM Tris, 150 mM NaCl, 10 mM MgCl_2_, 2 mM CaCl_2_). Cell pellets were resuspended in 1 mL of MP buffer containing phage at ≥10^10^ PFU mL^−1^ for a multiplicity of infection of 10. The cell/phage resuspension was incubated for 24 h at 37°C in atmospheric CO_2_. After 24 h, cells were resuspended by gently pipetting 10 times and serially diluted. Dilutions were plated on supplemented 7H10 agar plates containing kanamycin and various concentrations of POA. Colonies were counted at day 14 to assess POA susceptibility. A kanamycin-resistant empty vector strain (M. tuberculosis strain H37Rv pUMN002) was treated identically with MP buffer containing no phage for comparison as a vehicle control.

### Construction of M. tuberculosis H37Rv deletion mutant strains.

Deletion of *aceE*, *pckA*, and *rseA* in M. tuberculosis H37Rv was accomplished using the ORBIT recombineering system (40). In brief, M. tuberculosis H37Rv cells previously transformed with pKM461, encoding tetracycline-inducible Che9c RecT annealase and Bxb1 integrase, were grown to mid-log phase (OD_600_ ≈ 0.8) in supplemented 7H9 medium containing 50 μg mL^−1^ kanamycin for selection of pKM461-containing cells. Once an OD of ∼0.8 was reached, anhydrotetracycline was added to a final concentration of 500 ng mL^−1^ and incubated for 8 h. Following induction, 2 M glycine was added to the culture and incubated further overnight (∼16 h) while being shaken at 37°C. The next day, cells centrifuged in-50 mL conical tubes at 4,300 rpm for 10 min to pellet cells. Cell pellets were resuspended in an equal volume of 10% glycerol. The centrifugation and washing steps were repeated. A final centrifugation and washing step were performed, with cells being resuspended in 3 mL of 10% glycerol. Cells were electroporated with 1 μg of a targeting oligonucleotide containing an *attP* site core and 200 ng of the knockout plasmid pKM464 carrying an *attB* site and hygromycin resistance marker. The targeting oligonucleotide sequence for deletion of *aceE* was CCCGACCGAGTTCGGGTGATCCGCGAGGGTGTGGCGTCGTATTTGCCCGACATTGATCCCGGTTTGTCTGGTCAACCACCGCGGTCTCAGTGGTGTACGGTACAAACCCAGACCACGGATCCCGGTCCCGGGGCCTAACGCCGGCGAGCCGACCGCCTTTGGCCGAAT, that for deletion of *pckA* was TGCGTGCGGGGGCTTATGCGTCTGCTCGCCCTAACCTAGGCGCTCCTTCAGGGCGTCGAAGGTTTGTACCGTACACCACTGAGACCGCGGTGGTTGACCAGACAAACCATCCAGACCGGGGATGGTCGCTGAGGTCATCGAATTCTCCTGCGTAGTTATCGGGTGCTC, and that for deletion of *rseA* was GCAGCAACCCCGCCATGCGCTGCGACAAGTGGCTCACCGGCTAGCGACGCACCCGCGATTGCCGGCCCCGGGTTTGTACCGTACACCACTGAGACCGCGGTGGTTGACCAGACAAACCCACATGTCCCACGCTTCCGGGGTCGGCCATCACCACCTCCTTCCGCCACCTAGCGAGCCACCGGTATCTC. The Bxb1 *attB* site is underlined. Transformants were recovered in 2 mL of supplemented 7H9 and shaken overnight at 37°C. The following day, cells were plated on supplemented 7H10 medium containing 50 μg mL^−1^ hygromycin to select for integration of pKM464 and 2% sucrose for curing of the recombineering plasmid pKM461, which encodes the *sacB* counterselectable marker. Deletion strains were confirmed by PCR and sequencing of the chromosome-pKM464 junction.

### Analysis of *sigE* mutations in clinical strains.

To identify potential PZA resistance signatures of selection on SigE, we contrasted mutation frequency in two sets of isolates. The first set of isolates was a global set of background isolates derived from the genome-wide Mycobacterium tuberculosis variation database (71), with 224 PZA-resistant (PZA-R) and 766 PZA-susceptible (PZA-S) isolates, and the Global Consortium for Drug-resistant Tuberculosis Diagnostics (72), with 235 PZA-R and 80 PZA-S isolates. The second set of isolates was a curated set of PZA-monoresistant isolates (*n *= 18).

### Evaluation of drug interactions using checkerboard assays.

Drug interactions were evaluated through standard checkerboard assays. Briefly, supplemented Middlebrook 7H9 medium (pH 6.6) was used to culture M. tuberculosis H37Rv, M. tuberculosis Erdman, or M. tuberculosis H37Rv Δ*sigE* to late log phase. Strains were subcultured to an OD_600_ of 0.01 into 5 mL of supplemented Middlebrook 7H9 (pH 6.6). Bottles were arrayed into rows and columns. PZA was added to each row using a log_2_ dilution scheme from 1,600 μg mL^−1^ to 50 μg mL^−1^ and a no-drug control. The second drug was subsequently added in a log_2_ dilution scheme to each column. The drug concentration ranges tested against M. tuberculosis H37Rv were as follows: meropenem, 2 μg mL^−1^ to 0.125 μg mL^−1^; EMB, 0.5 μg mL^−1^ to 0.0625 μg mL^−1^; INH, 30 ng mL^−1^ to 7.5 ng mL^−1^; and d-cycloserine, 10 μg mL^−1^ to 1.25 μg mL^−1^. The range for meropenem against M. tuberculosis Erdman was 1 μg mL^−1^ to 0.0625 μg mL^−1^. Bottles were incubated at 37°C, and the OD_600_ was measured after 7 days of incubation. For checkerboard assays, the MIC was defined as the minimum concentration of drug required to inhibit at least 50% of growth relative to the drug-free control. Fractional inhibition concentration index (FICI) was calculated using the following formula: [(MIC of drug B in the presence of a given concentration of drug A)/(MIC of drug B alone)] + [(MIC of drug A in the of presence of drug B)/(MIC of drug A alone)]. If the highest concentration of a drug did not inhibit growth by 50%, the MIC_50_ value used in the FIC calculation was set as 2× the highest concentration that was tested. Drug interactions were defined as follows: FICI values of ≤0.5 were considered synergistic, FICI values of >0.5 but ≤1.0 were considered additive, FICI values from 1.0 to 4.0 were considered indifferent, and FICI values of >4.0 were considered antagonistic (73).

### Data availability.

All relevant data are available upon request.
