# Supplementary material for: Pyrazinamide Susceptibility Is Driven by Activation of the SigE-Dependent Cell Envelope Stress Response in Mycobacterium tuberculosis
Source: mBio. 2022 Feb 1;13(1):e00439-21. doi: 10.1128/mbio.00439-21 (PMC8805019; doi:10.1128/mbio.00439-21)
Supplement: TABLE S1 [file mbio.00439-21-st001.docx]

| **Table S1.** POA susceptibility of *M. tuberculosis* and *M. bovis* is enhanced by infection with mycobacteriophage phAE180 similar to exposure to low pH. | | | | | |
| --- | --- | --- | --- | --- | --- |
| Strain | pH 6.6 | pH 5.8 | | pH 6.6 with phage phAE180 infection | |
|  | POA MIC^a^  (µg ml^-1^) | POA MIC (µg ml^-1^) | Fold decrease in MIC^b^ | POA MIC  (µg ml^-1^) | Fold decrease in MIC^b^ |
| *M. tuberculosis* H37Rv | 200 | 50^31^ | 4 | 50 | 4 |
| *M. bovis* BCG Pasteur | 1200 | 100^31^ | 12 | 75 | 16 |
| ^a^MIC (minimum inhibitory concentration) is defined as the minimum amount of drug to inhibit visible growth on 7H10 medium within 14 days. ^b^Fold decrease in MIC relative to MIC at pH 6.6 using non-phage infected bacilli. | | | | | |
