# Supplementary material for: Pyrazinamide Susceptibility Is Driven by Activation of the SigE-Dependent Cell Envelope Stress Response in Mycobacterium tuberculosis
Source: mBio. 2022 Feb 1;13(1):e00439-21. doi: 10.1128/mbio.00439-21 (PMC8805019; doi:10.1128/mbio.00439-21)
Supplement: TABLE S2 [file mbio.00439-21-st002.docx]

| **Table S2.** Genotypic and phenotypic characterization of *M. tuberculosis* H37Rv pyrazinamide resistant transposon mutants | | | | | |
| --- | --- | --- | --- | --- | --- |
| Strain  (transposon insertion site) | Relevant features | Agar Medium | Liquid Medium | | |
|  |  | POA MIC*^a^* (µg ml^-1^) | POA MIC_90_*^b^* (µg ml^-1^) | PZA MIC_90_ (µg ml^-1^) | INH MIC_90_ (µg ml^-1^) |
| H37Rv | Parental strain | 200 | 200 | 50 | 0.0625 |
| *pckA*::*Tn*  (252157) | Disruption of gene for PEP carboxykinase | 3200 | 400-800 | 100 | ≤0.0625 |
| *panD*::*Tn*  (4043886) | Disruption of gene for L-aspartate alpha-decarboxylase | 800 | >1600 | 100 | ≤0.0625 |
| *gabP*::*Tn*  (612955) | Disruption of gene for GABA permease | 800 | 800 | 100 | ≤0.0625 |
| *Rv2705c*::*Tn*  (3020165) | Disruption of gene for hypothetical protein | 400 | 400-800^1^ | 100^1^ | ≤0.0625^1^ |
| *Rv2706c*::*Tn.1^c^*  (3020304) | Disruption of gene for hypothetical protein | 200 | 400 | 100 | ≤0.0625 |
| *Rv2706c*::*Tn.2^c^*  (3020355) | Disruption of gene for hypothetical protein | 400 | 800^1^ | 100^1^ | ≤0.0625^1^ |
| *Rv3256c*::*Tn*  (3636565) | Disruption of gene for hypothetical protein | 400 | 800^1^ | 50-100^1^ | ≤0.0625^1^ |
| P*_clpC1_*::*Tn*  (4040795) | Insertion in promoter region of *clpC1* | ND*^d^* | 400-800 | 50 | ≤0.0625 |
| *Rv3727*::*Tn*  (4174408) | Disruption of gene for hypothetical protein | 800 | 1600 | 100 | ≤0.0625 |
| P*_Rv3916c_*::*Tn*  (4405250) | Insertion in promoter region of gene for hypothetical protein | 400 | 800^1^ | 100^1^ | ≤0.0625^1^ |
| *^a^*MIC, defined as minimum amount of drug to inhibit visible growth within 14 days on 7H10 agar pH 6.6; *^b^*MIC_90_, defined as minimum amount of drug to inhibit 90% of growth relative to no drug control in 7H9 at pH 6.6 for POA and INH, and pH 5.8 for PZA; *^c^*denotes distinct mutants isolated with transposon insertions in different TA sites within the *Rv2706c* locus; *^d^*denotes not determined. ^1^Reported in Modlin SJ, Elghraoui A, Gunasekaran D, Zlotnicki AM, Dillon N, Dhillon N, Kuo N, Robinhold C, Chan CK, Baughn AD, Valafar F (2021) Structure-Aware Mycobacterium tuberculosis Functional Annotation Uncloaks Resistance, Metabolic, and Virulence Genes. mSystems 6:e0067321. | | | | | |
