# Supplementary material for: Pyrazinamide Susceptibility Is Driven by Activation of the SigE-Dependent Cell Envelope Stress Response in Mycobacterium tuberculosis
Source: mBio. 2022 Feb 1;13(1):e00439-21. doi: 10.1128/mbio.00439-21 (PMC8805019; doi:10.1128/mbio.00439-21)
Supplement: TABLE S3 [file mbio.00439-21-st003.docx]

| **Table S3.** Fold change in relative abundance of *himar1* insertions in *M. tuberculosis* loci following selection on medium containing POA | | |
| --- | --- | --- |
| **Gene or locus** | **log_2_ fold change in relative abundance^a^** | **negative log_10_ *p* value*^b^*** |
| *MTB000001*_(*ileT*)_promoter | 7.56243195 | 0.59829795 |
| *Rv0066c*_(*icd2*) | 1.19872501 | 0.221444893 |
| ***Rv0211*_(*pckA*)** | **9.938975945** | **5.542063907** |
| ***Rv0495c*** | **7.417876232** | **4.579635373** |
| *MTB000115*_(*ncRv10666*) | 1.940967836 | 0.727939546 |
| *Rv0668*_(*rpoC*) | 8.445373151 | 0.458274438 |
| *Rv0818* | 0.421865474 | 0.481253169 |
| *Rv0819*_(*mshD*) | 1.482220347 | 1.184549401 |
| ***Rv0820*_(*phoT*)** | **4.344184849** | **3.035797579** |
| *Rv0823c* | -1.44953044 | 0.812740805 |
| *MTB000010*_(*lysT*)_promoter | 3.444040285 | 0.720607621 |
| ***Rv0928*_(*pstS3*)** | **3.883024448** | **3.55623194** |
| ***Rv0929*_(*pstC2*)** | **3.867477466** | **3.151157088** |
| ***Rv0930*_(*pstA1*)** | **5.401490781** | **1.732973802** |
| ***Rv1080c*_(*greA*)** | **5.474208198** | **3.370200033** |
| *Rv1094*_(*desA2*) | 3.831642546 | 0.775973227 |
| ***Rv1099c*_(*glpX*)** | **1.054420572** | **1.893419606** |
| *Rv1197*_(*esxK*) | -0.872669311 | 1.167674771 |
| ***Rv1221* (*sigE*)** | **4.233984622** | **2.342157809** |
| *Rv1248c*_(*kgd*) | 1.240238357 | 1.017201584 |
| *Rv1296*_(*thrB*) | 7.29640042 | 0.359136055 |
| ***Rv1339*** | **4.874396133** | **4.920459234** |
| *Rv1539*_(*lspA*) | 4.418694654 | 1.229138949 |
| ***Rv1540*** | **8.222584168** | **3.57788002** |
| *Rv1595*_(*nadB*) | 12.38167752 | 1.060251027 |
| ***Rv1957*** | **4.542267335** | **3.102537059** |
| ***Rv2198c*_(*mmpS3*)** | **2.532860132** | **1.847315827** |
| *Rv2215*_(*dlaT*)_promoter | -0.783637471 | 1.038690275 |
| ***Rv2241*_(*aceE*)** | **5.136684128** | **9.674484676** |
| *Rv2347c*_(*esxP*) | -1.725395003 | 1.606177766 |
| *Rv2348c*_promoter | -1.398592551 | 0.763130912 |
| *Rv2357c*_(*glyS*) | 1.191599661 | 0.295464187 |
| ***Rv2397c*_(*cysA1*)** | **4.768809132** | **2.620144189** |
| ***Rv2398c*_(*cysW*)** | **3.550068641** | **1.906454967** |
| *Rv2399c*_(*cysT*) | 3.659539028 | 0.967335119 |
| ***Rv2400c*_(*subI*)** | **4.564997085** | **3.272202511** |
| *Rv2444c*_(*rne*) | 8.72391668 | 0.571827443 |
| *Rv2643*_(*arsC*) | 2.684124426 | 0.451764333 |
| *MTB000035*_(*valT*)_promoter | 0.617689008 | 0.081357091 |
| ***Rv2690c*** | **8.350806576** | **5.538845712** |
| *Rv2691*_(*ceoB*) | -0.721782179 | 0.62674835 |
| *Rv2705c* | 2.329437308 | 0.305125651 |
| *Rv2706c* | 10.93938972 | 0.78006675 |
| *Rv2840c* | 8.950225481 | 0.892415351 |
| *Rv2940c*_(*mas*)_promoter | 0.720290494 | 0.463990742 |
| *Rv2987c*_(*leuD*) | 5.466801297 | 0.401735741 |
| *Rv3024c*_(*trmU*) | 4.864855294 | 0.870819402 |
| *Rv3200c* | -1.272703701 | 0.428295479 |
| ***Rv3256c*** | **9.977752378** | **1.887753882** |
| *Rv3320c* | 2.95716587 | 0.385147006 |
| *Rv3596c*_(*clpC1*)_promoter | 5.792972592 | 0.298814825 |
| *Rv3601c*_(*panD*) | 9.137511322 | 0.981601056 |
| ***MTB000159*_(*ncRv13660c*)** | **7.862580188** | **1.935959704** |
| *Rv3676*_(*crp*) | 7.982340377 | 0.792577558 |
| *Rv3680* | 2.070014041 | 0.913097741 |
| *Rv3729* | -0.572514158 | 0.101110981 |
| *Rv3810*_(*pirG*) | 0.457920374 | 0.286381326 |
| *Rv3916c*_promoter | 5.35757514 | 0.684587343 |
| *^a^*Fold change in relative abundance of *himar1* insertions per gene was calculated from Dataset S1. The sum relative read abundance from TA sites with ≥5 read counts following selection on POA was divided by the sum relative read abundance of the corresponding sites from the no POA condition. ***^b^****p* values were calculated using a two-tailed paired *t*-test. Values shown are negative log_10_ transformed. Genes showing greater than 1 log_2_-fold enrichment in the presence of POA with a negative log_10_ *p* value of >1.3 are marked in bold. | | |
