# Supplementary material for: Pyrazinamide Susceptibility Is Driven by Activation of the SigE-Dependent Cell Envelope Stress Response in Mycobacterium tuberculosis
Source: mBio. 2022 Feb 1;13(1):e00439-21. doi: 10.1128/mbio.00439-21 (PMC8805019; doi:10.1128/mbio.00439-21)
Supplement: FIG S1 [file mbio.00439-21-sf001.pdf]

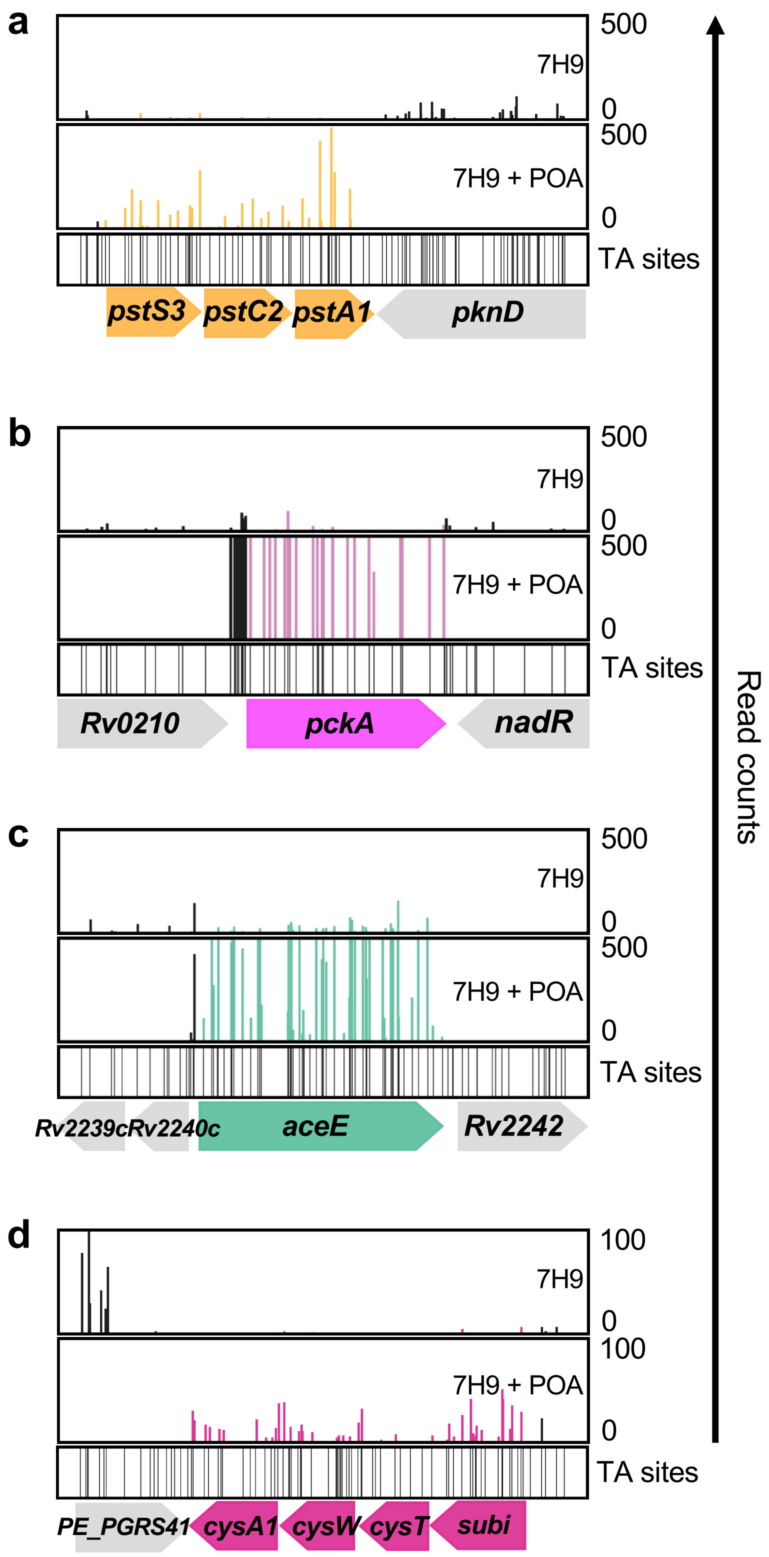

Figure S1. Genes associated with *M. tuberculosis* PZA susceptibility by TnSeq. Libraries of 2x10^5 independent *himar1* mutants (4-fold saturation) were plated on 7H9 agar without (top panels, 7H9) or with POA (middle panels, 7H9 + POA). Genomic DNA was extracted, processed and sequenced as described in Minato, *et al* 2019 *mSystems*. Bottom panels show all TA dinucleotides of the region that is illustrated. Mean read count comparisons from two independent replicates are shown for *pstS3C2A1* (**a**) *pckA* (**b**), *aceE* (**c**), and *subI-cysTWA1* (**d**).
